# 

**Published:** 1893-11

**Authors:** 


### CONTENTS:

Article I.—Improvements in Dentistry. By F. J. S. Gorgas, M. D., D. D. S. - - - - - - - 289

" II.—Alabama Dental Association. By Mrs. J. M. Walker. 294

" III.—Pulpless Teeth also Subject to Erosion. By J. M. Howe. - - - - - - - 304

" IV.—Manipulating Oxyphosphate of Zinc as a Filling-Material. By H. G. Register. - - - - 306

" V.—Trichloracetic Acid in Pyorrhea. - - - 308

" VI.—The Progress of Dentistry. - - - - 313

" VII.—What the Hiccough is. - - - - - 314

" VIII.—Filling Roots with Gutta-Percha Dissolved in Chloroform. By Dr. R. I. Blakeman. - - - 317

" IX.—New Remedies and their Application. By L. P. Bethel, M. D., D. D. S. - - - - - 320

" X.—A Brunswick Stew. By Dr. C. A. Rominger. - 325

### MONTHLY SUMMARY.

Nervous Exhaustion. - - - - - - - - 329

The Bridge-work Fad. - - - - - - - 330

Chas. Elroy on How to Treat Cocaine Poisoning. - - - 331

Foreign Diplomas in Great Britain. - - - - - 332

F. Peterson on Clonic Spasm of the Muscles of Mastication. - 333

Phagocytosis. - - - - - - - - - 334

Mr. Humby. - - - - - - - - - 335

It is the Opinion of Dr. Rhein. - - - - - - 335

Eucalyptol. - - - - - - - - - 336

W. K. Vanderbilt. - - - - - - - - 336

Many a Child in After Years. - - - - - - 336

The Last Straw. - - - - - - - - 336

